# Early postoperative compilations of bone filling in curettage defects

**DOI:** 10.1186/s13018-019-1297-4

**Published:** 2019-08-16

**Authors:** Clark J. Chen, Earl W. Brien

**Affiliations:** 1 Miller School of Medicine, University of Miami, Miami, FL 33136 USA; 2 Department of Orthopaedic Surgery, Cedars Sinai Medical Center, Los Angeles, CA 90048 USA

**Keywords:** Curettage, Benign bone tumor, Giant cell tumor, Bone filling, Autograft, Allograft, Bone graft substitute, Polymethyl methacrylate, Postoperative fracture

## Abstract

**Background:**

Curettage is widely used in orthopedic oncology; the defect created frequently requires filling for mechanical and functional stability for the bones and adjacent joint. Allograft, bone graft substitute, and polymethyl methacrylate (PMMA) are the most common substances used each with their benefits and drawbacks. The aim of the study is to show that good functional result can be achieved with curettage and bone filler, regardless of type.

**Methods:**

A series of 267 cases were reviewed between 1994 and 2015 who received curettage treatment and placement of a bone filler. Endpoints included fracture, infection, cellulitis, pulmonary embolism, and paresthesia. Complication rates at our single institution were compared against literature values for three study cohorts: allograft, bone graft substitute, and PMMA bone fillers. Friedman test, Wilcoxon test, and *Z*-score for two populations were used to compare our subset against literature values and between different bone filling types.

**Results:**

Our cases included 18 autografts, 74 allografts, 121 bone graft substitute, and 54 PMMA of which the bulk of complications occurred. Our overall complication rate was 3.37%. Allograft has a complication rate of 1.35%, bone graft substitute of 4.13%, and PMMA of 5.56%. Other techniques did not yield any complications. Combination filling techniques PMMA + allograft and PMMA + bone graft substitute had sample sizes too small for statistical comparison. Statistical comparison yielded no significant difference between complications in any of the filling groups (*P* = 0.411).

**Conclusions:**

Some has even argued that bone defects following curettage do not require bone filling for good outcome. However, many structural or biologic benefits that aid in earlier return to functionality can be conferred by filling large bone defects. There was no significant difference in postoperative complication rates between allograft, bone graft substitute, and PMMA when compared at our institution and with literature values. Nevertheless, one complication with a large defect filled with allograft, requiring a subsequent reconstruction using vascularized fibular graft. Taking everything into account, we see bone graft substitute as a suitable alternative to other bone filling modalities.

## Introduction

Curettage is widely used in orthopedic oncology used to treat benign, aggressive, and in some cases metastatic bone lesions [1]. The defect created from intralesional curettage frequently requires defect filling to impart mechanical and functional stability to the treated bone and adjacent joint [2, 3].

Allograft, bone graft substitute, and polymethyl methacrylate (PMMA) are common substances used for filling the boney defect, each with their advantages. The gold standard that has widely been accepted is the allograft method [4]. It is thought that the tissue is better integrated into the host’s body, offering higher biocompatibility [5]. PMMA bone cement has been an alternative to the costly allograft and has widely been compared against allografts in the past decade [6]. The newer brand of bone filler is the bone substitute category which contains a larger range of synthetic substances from calcium phosphate or sulfate mixtures to bioengineered matrixes [7, 8]. A relatively novel technique in which PMMA is added to autograft or allograft has also seen promising results at providing mechanical stability in more recent years [9, 10]. Short-term complications after bone filling can lead to a difficult course of recovery requiring surgical revision; these include recurrence, fracture, or deep infection. The aim of the study was to support our hypothesis that good functional result can be achieved with curettage and bone filler, regardless of bone filler type. Depending on the specific type of tumor, other adjuvant therapies such as liquid nitrogen or phenol are also used. Although, adjuvant therapy may impact bone quality and bone formation while large defects may impact normal weight bearing, which can subsequently lead to a higher risk for fracture, independent of bone filling techniques, they will not be discussed in this paper.

## Materials and methods

We retrospectively reviewed charts to in patients who had been treated with curettage and a type of bone filler between 1994 and 2015. Patients were filtered based on a series of inclusion and exclusion criteria to limit the scope of the study to early postoperative complications (Table 1). This included a spectrum of tumor types from benign tumors to giant cell tumors (GCTs). Complications were the main factor of this study, and endpoints included fracture, infection, cellulitis, pulmonary embolism, and paresthesia. Long-term clinical follow-up was done in all patients including imaging of the functional joint which allowed assessment of bone stability, to examine whether there had been healing or development of complications. Tumor reoccurrence and metastasis were not considered in this present study.

**Table 1:** Early postoperative complication inclusion and exclusion criteria

Inclusion	Exclusion
• Patients between 1994 to 2015 • All tumor types treated by curettage and bone filling • At least 2-year follow-up with imaging • Complications endpoint: ▪ Fracture ▪ Infection ▪ Cellulitis ▪ Pulmonary embolus ▪ Paresthesia or neuropraxia	• Bone graft substitute other than calcium phosphate or calcium sulfate • En bloc resection • Excluded complications: ▪ Metastasis ▪ Reoccurrence

The surgical technique involved curettage in all cases, and a proportion used a variety of additional adjuvant therapy, including liquid nitrogen cryotherapy. Bone filling type varied between autograft, allograft, bone graft substitute, PMMA, PMMA + allograft, and PMMA + bone graft substitute. At our institution, synthetic bone graft composed of porous β-tricalcium phosphate (β-TCP) (Vitoss Bone Graft Substitute) was used in the majority of cases. Complications were verified using radiographic evidence along with clinical assessment according to the records. Reoccurrence, while a complication related to graft type, was not the focus of this study and was not recorded as one of the short-term postoperative complications of this study.

**Fig. 1:**
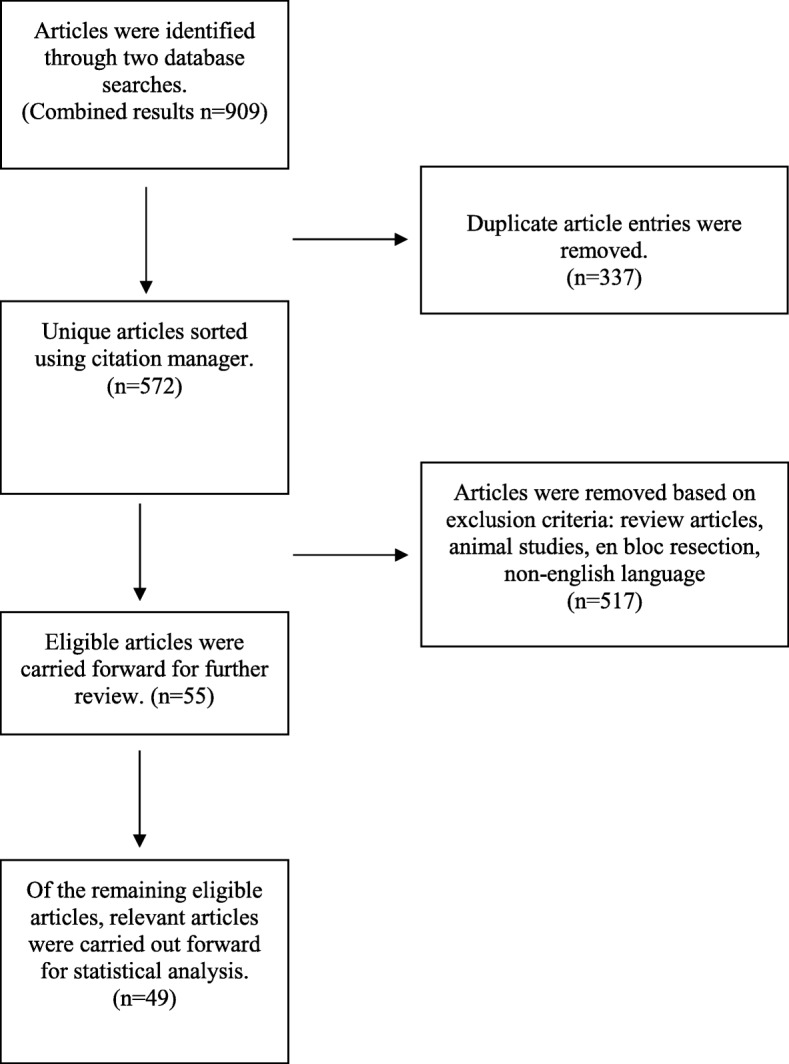
Study flow chart showing the review process of the literature to select the 49 articles used for the statistical comparison of this study

A search of the literature was done on Web of Science and Medline on January 2018. The search terms were as follows: (“curettage” AND “orthopaedic oncology” AND (“PMMA” OR “graft”) AND “complicat*”). The result was 909 articles, with 337 duplicate articles, resulting in 572 non-duplicate articles (Fig. 1). Articles were removed from consideration based on inclusion and exclusion criteria based on two independent reviewers; differences were settled by discussion of the two reviewers. Review articles, animal studies, and en bloc resection with graft reconstruction were not included in this study. For classification purposes of this paper, the two primary groups of bone graft substitute were calcium phosphate and calcium sulfate types. Under the umbrella of calcium phosphate, we included hydroxyapatite, hydroxyapatite, calcium phosphate cement, β-tricalcium phosphate, α-tricalcium phosphate, and tetracalcium phosphate. Calcium sulfate and its derivatives formed a separate group for complication rate analysis due to the nature of the graft. English language articles that met the inclusion and exclusion criteria yielded 10 autograft, 11 allograft, 17 bone graft substitute, 10 PMMA, and 1 PMMA + allograft articles were collected, and postoperative complication rates were extracted into their corresponding tables. Literature complication rates were calculated based on the same endpoint as our retrospective review. Complications were considered in a similar fashion in all selected articles, when that information was present.

Univariate statistical analysis was used when appropriate including, first, the Friedman rank test for non-parametric data to compare the different types of fillings. Then, the Wilcoxon signed-rank test was used to compare the indicated statistically relevant subset of the data for more specific analysis. A *Z*-score for two population proportions was used to compare a single categorical characteristic between two populations. In this study, *Z*-score for two population proportions was used to analyze and compare rates of complications of the population at our institution and compared against summed literature values. Statistical significance was set at (*P* < 0.05) for a difference in complication rates.

## Results

Two hundred sixty-seven cases were reviewed, with a majority of lesions ranging from solitary bone cysts (SBC), nonossifying fibromas (NOF), and fibrous dysplasia (FD), and giant cell tumor being the types of lesions. Our cases used 74 allografts, 121 bone graft substitute, and 54 PMMA of which the bulk of complications occurred. Other filling techniques PMMA + allograft, PMMA + bone graft substitute, and autograft totaled 18 out of 267 cases. Allograft has a complication rate of 1.35%, bone graft substitute had a complication rate of 4.13%, PMMA had a complication rate of 5.56%, and the other techniques did not yield any complications. Our total complication rate was 3.37% (Table 2). In the treatment of GCT, two cases of bone graft were used. Statistical analysis could not be done on PMMA with combination of allograft or bone graft substitute because of poor power in sample size.

**Table 2:** Short-term postoperative complications

Type of bone filling	Cases	Fracture	Infection	Cellulitis	PE^a^	Paresthesia	Rate (%)
Allograft	74	1	1	0	0	0	2.70
Bone graft substitute	121	1	1	1	1	1	4.13
Polymethyl methacrylate	54	1	2	1	0	0	7.41
PMMA + allograft	5	0	0	0	0	0	0.00
PMMA + bone graft substitute	3	0	0	0	0	0	0.00
Autograft	10	0	0	0	0	0	0.00
Total	267	3	4	2	1	1	*4.12*

^a^Pulmonary embolus

The results from the Friedman rank test showed no statistically significant difference (*P* = 0.411) between the complication rates between the bone filler groups. The Wilcoxon signed-ranked test was used to confirm no statistical significance between allograft, bone graft substitute, and PMMA (*P* > 0.05) in all three test combinations (Table 3). From the *Z*-score for two population proportions, there was no statistical difference when comparing our complication rates against literature rates (*P* > 0.05) across all types of bone filler (Table 4).

**Table 3:** Wilcoxon’s signed-rank test

	Bone graft substitute vs allograft	Bone graft substitute vs PMMA	Allograft vs PMMA
*P* value	0.214	0.221	0.109

Significance value set at *P* < 0.05

**Table 4:** *Z*-score for two population proportions

	Our rates (%)	*n*	Literature rates (%)	*N*	*Z*-score	*P* value
Allograft	2.70	74	3.24	401	− 0.244	0.810
Bone graft substitute						
Calcium sulfate			6.52	138	− 0.849	0.395
Calcium phosphate	4.13	121	3.48	661	0.355	0.719
All			4.21	950	− 0.040	0.968
PMMA	7.41	54	5.59	483	0.543	0.589

## Discussion

When confronted with a large bone filling defect after, it has been the standard to add filling to add structural support. Although it is the most biologically similar, the limited availability of autograft is a concern for large defects. Despite its availability, the risk of disease transmission associated with the use of allograft is not insignificant [11]. PMMA cement may provide instant stability, but is not a biologically integrated bone filler [12]. Many different bone substitutes have reached the market, designed toward filling these defects, but there is little evidence for their efficacy in vivo [13]. We noted several complications among the common bone filling modalities and sought out to investigate the functional outcome of curettage and bone filler type.

### Autograft

The most common autograft sites for curettage in the literature include the fibular graft and iliac plate graft. The majority of retrospective literature have shown no significant difference in bone incorporation between autograft and allograft [14, 15]. However, a few studies have reported radiographic evidence of improved consolidation of cyst in autograft filling in aneurysmal bone cyst after curettage [16]. For large lesions, a large amount of graft is difficult to harvest especially so in younger individuals and children due to concerns with quantity and interfering future skeletal growth [14]. In larger defects, it is common to mix autograft and allograft to fill curettage defects and has shown good results without increased risk of complication, but we did not use this combination in our case series [15, 17].

Our series had no complications associated with autograft filler, when compared against aggregate literature rates (2.43%) (Table 5). Complications of this group may not have been elucidated due to the small sample size in our series. Paresthesia was the most common complication in the literature for allograft filler (1.50%). We speculate that paresthesia was the most common because of the location of the lesions, primarily in the lower limb, leading to peroneal injury [21, 26].

**Table 5:** Literature review of autograft

Author	Year	Cases	Fracture	Infection	Cellulitis	PE	Parastesia/neuralpraxia	Other	Rate (%)
Georgiannos et al. [18]	2016	46	0	0	0	0	0	0	0.00
Chen et al. [19]	2015	15	0	0	0	0	0	0	0.00
Gouin et al. [20]	2013	17	0	0	0	0	0	0	0.00
Badekas et al. [21]	2013	58	0	0	0	0	1	0	1.72
Ulucay et al. [22]	2009	21	0	0	0	0	0	0	0.00
Yercan et al. [23]	2004	76	0	2	0	0	0	0	2.63
Lindner et al. [24]	2000	64	0	1	1	0	2	0	6.25
Gibbs et al. [25]	1999	24	0	0	0	0	0	0	0.00
Chiang et al. [26]	1995	8	0	0	0	0	1	0	12.50
Total		329	0	3	1	0	4	0	*2.43*
			0.00%	0.91%	0.30%	0.00%	1.22%	0.00%	

A common complication that was observed in the literature but not tabulated was reoccurrence of the lesion [20–22, 25]. Gouin et al. describe reoccurrence as the primary complication in GCT treated with autograft, or no bone filler [20]. Similarly, Gibbs et al. describe reoccurrence in 16% of his cases in the setting on aneurysmal bone cysts with a majority of them occurring in the female population [25]. Because of our low use of allografts in aggressive tumors, we did not see reoccurrence as an issue. However, reoccurrence was defined as outside of our scope of interest and was not considered toward the complication rate of this study.

### Allograft

**Fig. 2:**
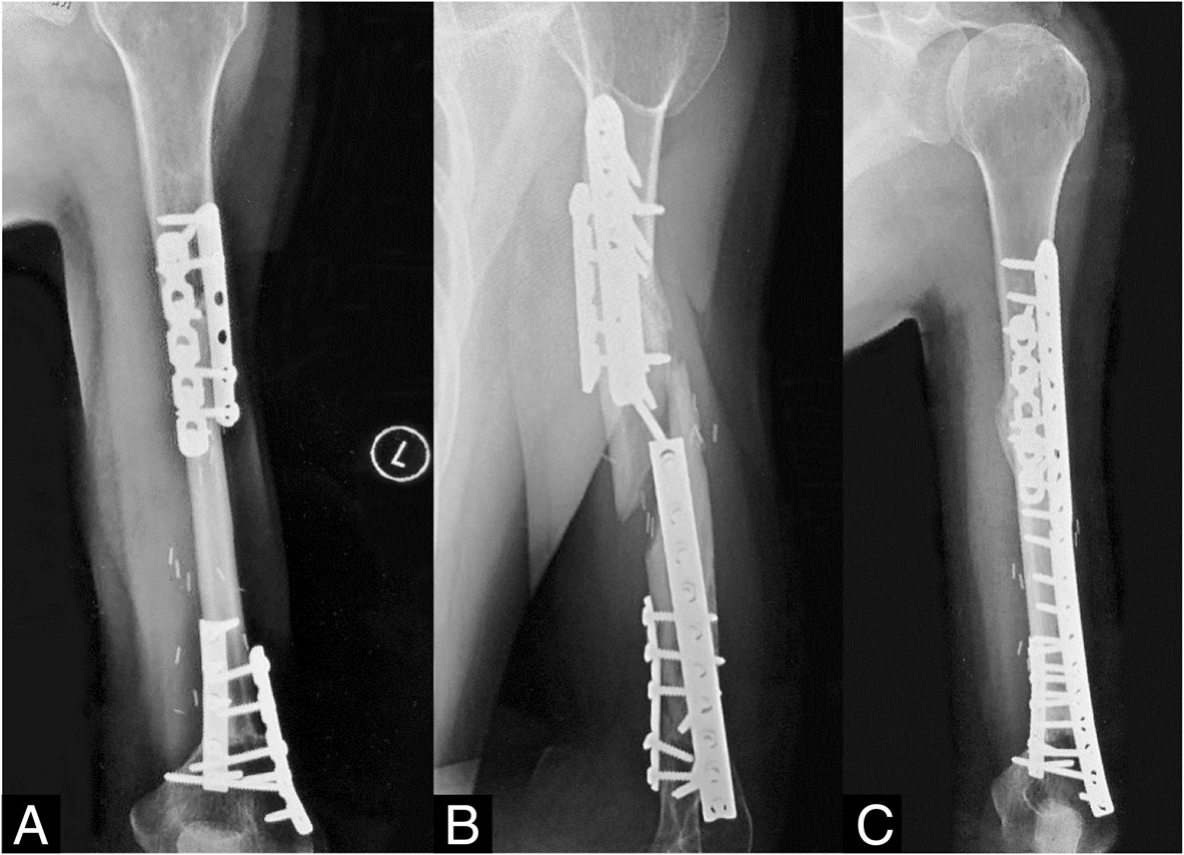
**a**–**c** Ewing’s sarcoma of the left humerus treated with chemotherapy, radiation, and resection. **a** Bulk allograft reconstruction showing ventral healing. **b** Fifteen years later, the patient suffered a fracture in his allograft from a twisting maneuver. **c** Because of the avascular central portion of the allograft, vascularized fibular autograft reconstruction was chosen, which healed well

Allograft is another commonly used bone filler that historically followed autograft use. Allograft is particularly an attractive option for filling large bone defects in children and adults. Its benefit as a calcium scaffold for new bone formation is comparable to that of autograft in several studies [14, 27]. Disadvantages include the risk of potential disease transmission and immunogenic risk from cadaveric bone. Allograft has a higher rate of mechanical failure and infectious complications reported in the literature compared to other defect filling techniques [28]. It leaves reasonable stability in cases of young active patients who will stress the graft and recipient region [29, 30]. Infection was the most deleterious complication that caused graft failure and required subsequent surgical revision [31]. Late fracture is also a risk when used as a large, intercalary segment and may require complex revision surgery and the conversion to a vascularized fibular graft because the viability of the allograft bone occurs only in close proximity to the union sites (Fig. 2).

In our series, there was a low rate of postoperative complications (2.70%), which were relatively comparable against our other bone filling groups and lower than the reported literature rates (3.24%) (Table 6). There was one case of postoperative infection in this group. Allograft was the lowest complication rate out of our three Wilcoxon signed-rank test comparison groups, but there was no statistically significant difference in complication rates compared to PMMA or bone graft substitute. Similarly in the literature, when allograft is compared against bone graft substitute, allograft has a longer healing time with similar complication profiles [33]. When comparing our complication rates against literature values, there was no significant difference between proportions (*P* = 0.8103).

**Table 6:** Literature review of allograft

Author	Year	Cases	Fracture	Infection	Cellulitis	PE	Parastesia/neuralpraxia	Other	Rate (%)
Kang et al. [32]	2015	34	0	0	0	0	0	0	0.00
Yang et al. [33]	2014	50	0	1	0	0	0	2^a^	6.00
Gouin et al. [20]	2013	62	0	0	0	0	0	0	0.00
Moretti et al. [29]	2011	30	1	0	0	0	0	0	3.33
Kim et al. [5]	2011	28	1	1	0	0	0	0	7.14
Lackman et al. [34]	2009	6	0	0	0	0	0	0	0.00
Ayerza et al. [35]	2009	22	0	0	0	0	0	1^b^	4.55
Yercan et al. [23]	2004	15	0	0	0	0	0	0	0.00
Lindner et al. [24]	2000	33	0	1	1	0	0	0	6.06
Shih et al. [36]	1998	104	0	0	0	0	0	1^c^	0.96
Sethi et al. [37]	1993	17	0	3	0	0	0	0	17.65
Total		401	2	6	1	0	0	4	*3.24*
			0.50%	1.50%	0.25%	0.00%	0.00%	1.00%	

^a^2 cases of non-union

^b^1 case of subchondral collapse

^c^1 case of avascular necrosis

The most common cause of complication in the literature was deep infection which occurred in our series once [37]. The occurrence of these reported infections occurred in the early adoption of using allograft for curettage defects in the 1990s, before current federal regulations and industry standards on processing and improvement quality control improvements by the FDA. Two cases of non-union was reported in the series which occurred in the lower extremity and humerus described by Yang et al. [33]. Additionally, there was a wider variety of complications, which was arranged into the “other” category in our table. One incidence of avascular necrosis and one incidence of subchondral collapse occurred in the literature [35, 36]. In the episode of avascular necrosis, both the multiple episodes of instrumentation and the avascular nature of allograft may have led to vascular injury at the proximal femoral neck [36]. The episode of subchondral collapse occurred at the proximal tibia where morselized allograft was used to fill the thinned subchondral region. There may be an intraoperative cause during the index operation, the patient may have been obese, or the joint may have been mechanically overloaded by the patient [35]. Although these complications were rare, both occurred lower extremity curettage, the first near the greater trochanter and the second near the distal articular surface of the femur.

### Bone substitute

Bone substitutes are among the newest and most varied type of bone filler available on the market. The main types include hydroxyapatite, calcium sulfate, calcium phosphate, calcium carbonate, β-tricalcium phosphate, and others. However, with regard to postoperative complications, the literature has shown acceptable complication rates in filling boney defects after curettage. Fracture and infection, including cellulitis, were the most concerning complications that commonly occurred, despite good functional outcome [38–40].

In our series, complication rate was low (4.13%), but comprised of 1 fracture, 1 infection, 1 cellulitis, 1 pulmonary embolus, and 1 paresthesia. Our rates were comparable to the literature rates of 4.21% for bone graft substitute (Table 7). There was one case of postoperative pulmonary embolus; we did not suspect that bone graft substitute was a causative factor. We attributed the incidence of pulmonary embolus to having the bulk of our case series being bone graft substitute (121/267) compared to other filler groups. With regard to the single fracture complication, the patient suffered the fracture while rock climbing 3 weeks postoperatively. This was treated conservatively and healed without complications or additional surgery.

**Table 7:** Literature review of bone graft substitute

Author	Year	Cases	Fracture	Infection	Cellulitis	PE	Parastesia/neuralpraxia	Other	Rate (%)	Type
Takeuchi et al. [41]	2018	26	1	1	0	0	0	0	7.69	Calcium phosphate
Rajeh et al. [42]	2017	8	0	0	0	0	0	0	0.00	Calcium phosphate
Rosario et al. [43]	2017	12	0	0	0	0	0	0	0.00	Calcium phosphate
Sakamoto et al. [44]	2017	4	0	0	0	0	0	0	0.00	Calcium phosphate
Guida et al. [7]	2016	116	0	0	3	0	0	0	2.59	Calcium phosphate
Damron et al. [45]	2013	55	0	0	0	0	0	0	0.00	Calcium phosphate
Gentile et al. [46]	2013	16	0	0	0	0	0	0	0.00	Calcium phosphate
Seto et al. [47]	2013	34	0	0	0	0	0	0	0.00	Calcium phosphate
Reppenhagen et al. [48]	2012	51	0	0	1	0	1	1^a^	5.88	Calcium phosphate
Van Hoff et al. [49]	2012	29	0	0	0	0	0	1^b^	3.45	Calcium phosphate
El-Adl et al. [50]	2009	34	0	2	1	0	0	0	8.82	Calcium phosphate
Hirata et al. [51]	2006	53	0	0	0	0	0	0	0.00	Calcium phosphate
Matsumine et al. [52]	2006	56	1	0	1	0	0	0	3.57	Calcium phosphate
Ogose et al.[53]	2005	53	4	0	0	0	0	0	7.55	Calcium phosphate
Yamamoto et al. [54]	2000	75	2	0	0	0	0	0	2.67	Calcium phosphate
Nicholas et al. [55]	1994	18	1	0	1	0	0	0	11.11	Calcium phosphate
Capanna et al. [56]	1991	21	0	1	0	0	0	0	4.76	Calcium phosphate
Andreacchio et al. [8]	2018	9	0	0	0	0	0	0	0.00	Calcium sulfate
Muramatsu et al. [57]	2014	5	0	0	0	0	0	1^c^	20.00	Calcium sulfate
Yang et al. (32)	2014	50	0	0	2	0	0	0	4.00	Calcium sulfate
Kim et al. [5]	2011	28	2	0	0	0	0	0	7.14	Calcium sulfate
Hou et al. [58]	2010	31	1	0	0	0	0	0	3.23	Calcium sulfate
Kelly and Wilkins [59]	2004	15	1	1	1	0	0	0	20.00	Calcium sulfate
Nakamura et al. [60]	2016	33	0	1	1	0	0	0	6.06	Calcium sulfate + tricalcium phosphate
Auston et al. [61]	2015	87	1	0	0	0	0	0	1.15	Calcium sulfate + tricalcium phosphate
Friesenbichler et al. [62]	2014	31	0	0	0	0	0	5^d^	16.13	Calcium sulfate + tricalcium phosphate
Total		950	14	6	11	0	1	8	*4.21*	
			1.47%	0.63%	1.16%	0.00%	0.11%	0.84%		

^a^1 case of CPRS

^b^1 case of avascular necrosis

^c^1 case of CPRS

^d^3 cases of aseptic skin inflammation, 2 cases of soft tissue cystic inflammation

In the literature, β-TCP as a component of bone substitute has been increasingly studied because of its biologic profile that could augment bone formation [47, 51, 62]. In trials against demineralize bone matrix, calcium bone filler had comparable rates for complication with overall good results [5]. However, not all combinations produced acceptable results; one study using a combination of β-TCP and calcium sulfate produced extreme inflammation at the implant site causing deleterious effects to the skin and soft tissue [60, 62]. In a subset of calcium sulfate complication rates (6.52%) compared to the calcium phosphate rates (3.48%) in the literature, it showed an almost double complication rate with a profile consisting of mainly of fracture and cellulitis. There was no significant difference between complication rates between our cases and literature values. When comparing the rates between literature calcium sulfate and calcium phosphate groups, there was a trend toward increased complications. We did observe prolonged serous drainage in the calcium sulfate-based graft and discontinued using this graft shortly thereafter. However, there was no statistical significance between groups (*P* = 0.09692). Further studies are required to determine if it is secondary to the increased inflammatory response from the calcium sulfate-based graft or possibly its carrier.

In more recent years, the literature has shifted toward favoring newer forms of calcium phosphate bone substitutes for their osteointegrative effects with a better side effect profile compared to other bone filling modalities. However, fracture (1.36%) and infection (1.06%) were still the leading cause of complication in our reviewed calcium phosphate subset. Other complications that were notable included long-term joint stiffness requiring early-stage physical therapy to combat and two cases of complex regional pain syndrome [42, 43]. A possible cause for these complications might be related to limiting activities immediately postoperatively or extruded graft outside of the boney defect causing additional soft tissue inflammation.

**Fig. 3:**
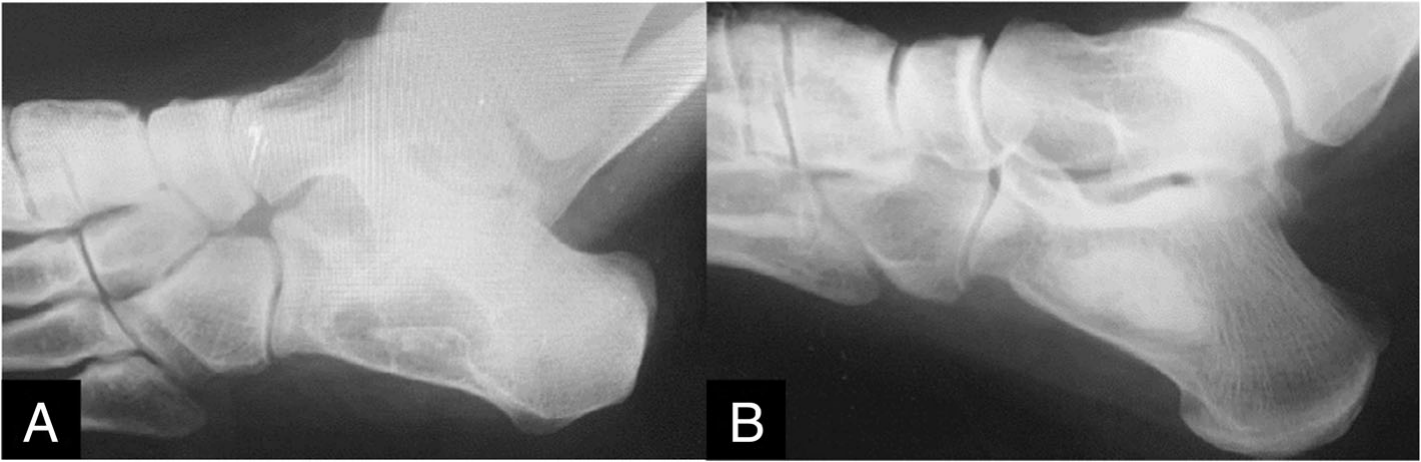
**a** A 27-year-old patient with lipoma of the calcaneus who received curettage of the lesion. **b** Following treatment with calcium phosphate, showing ventral incorporation at 18 months postoperatively

In our series, there were two cases of GCT that were treated with calcium sulfate bone graft that returned poor results. One case had a humerus fracture, and one case of infection. The fracture was treated with fracture brace and observation, and the infection was treated with irrigation and debridement, early range of motion, and late weight bearing. Our other cases using bone graft substitute largely healed uneventfully and showed long-term osteointegration (Fig. 3).

There have been several studies involving the combination use of bone substitute with allograft and PMMA, but they are few and scarce in case studies. Small case series involving bone graft substitute and allograft were described as successful options, but their effectiveness remains ambiguous because of the small sample size and lack of testing across centers [17, 63]. Likewise, the small sample size from our bone substitute combination subset creates difficulties to accurately compare their complication rates against our other subset complication rates. A combination of filler modalities may confer benefits of both graft types, but this requires further investigation.

### Polymethyl methacrylate

Curettage and PMMA cementation, used by Malawer and Marcove in the treatment of GCT, suggested an adjuvant aspect of PMMA bone cement, which offers local tumor necrosis of at least 1.5 mm into the cavity at > 100 °C [1, 64, 65]. The bulk of studies have been in GCT in our literature review, but chondrosarcoma, enchondroma, and FD also made appearances. Apart from its adjuvant nature in preventing reoccurrence in GCTs, PMMA can grant immediate benefits including mechanical strength and early recovery of weight bearing [6].

In our PMMA series, we had a complication rate of 7.41%, which was the highest complication rate out of all other filler groups, with infection and cellulitis being the main causes complications. This was constant with the literature rates (5.59%) (Table 8). However, a high rate of fracture rate existed in reported cases (4.14%) which is contrary to conventional thought given that PMMA allows for early weight bearing [9, 66, 69].

**Table 8:** Literature review of polymethyl methacrylate

Author	Year	Cases	Fracture	Infection	Cellulitis	PE	Parastesia/neuralpraxia	Other	Rate (%)
Benevenia et al.[9]	2017	22	5	0	0	0	0	0	22.73
Xing et al. [66]	2013	134	5	0	0	0	0	0	3.73
van der Heijden et al. [67]	2013	53	2	1	0	0	0	1^a^	7.55
Gouin et al. [20]	2013	94	0	0	0	0	0	0	0.00
Di Giorgio et al. [68]	2011	23	3	0	0	0	0	0	13.04
Gaston et al. [69]	2011	84	4	2	0	0	0	1^b^	8.33
Wada et al. [70]	2002	15	1	0	0	0	0	0	6.67
Bickels et al. [71]	2002	13	0	0	0	0	0	0	0.00
Yildiz et al. [72]	2001	12	0	1	0	0	0	0	8.33
Lindner et al. [24]	2000	33	0	0	0	0	1	0	3.03
Total		483	20	4	0	0	1	2	*5.59*
			4.14%	0.83%	0.00%	0.00%	0.21%	0.41%	

^a^1 case of pseudoarthrosis

^b^1 case of neuroma

**Fig. 4:**
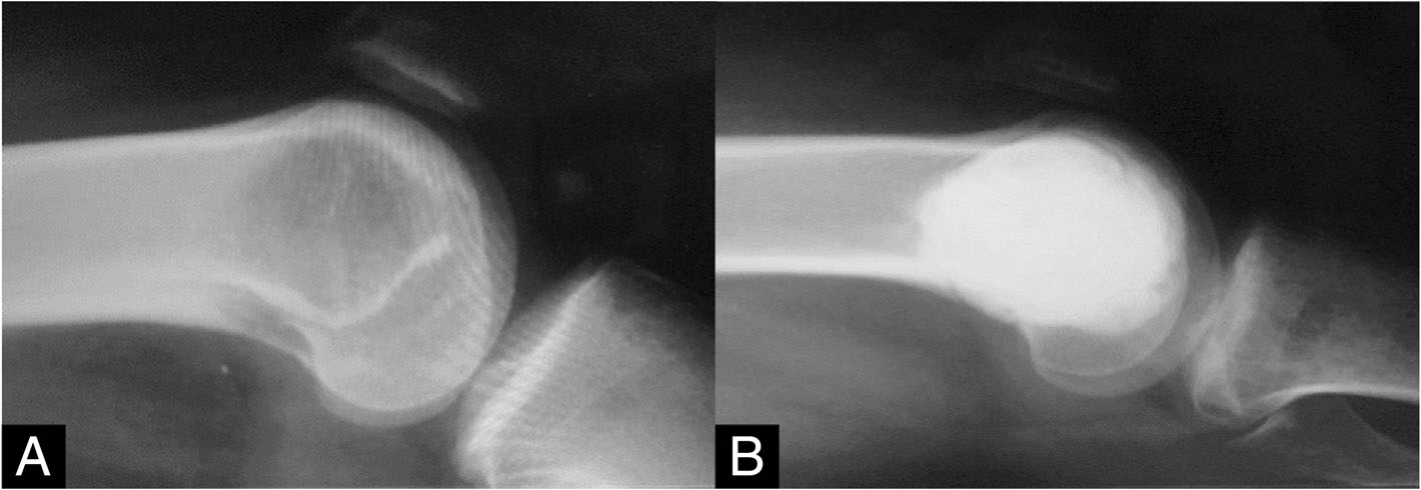
**a** A 23-year-old patient with a lytic lesion at distal femur. **b** Lesion treated with curettage and cementation, showing healing

We speculated that the high rate of fracture was caused by joint degeneration and joint stiffness which lends itself to early postoperative complications when used at the near the surface of a joint; PMMA may cause thermal injury and damage chondrocytes [67, 73]. This may trade off the ability for weight bearing with longer-term consequences particularly detrimental risks in children and adolescents. A large volume of PMMA cement produces a wide radiolucent region which may contribute to an interface between bone and cement that increases the risk of fracture (Fig. 4) [9, 70]. Paresthesia and nerve palsy are another complication described in PMMA, which may derive from local thermal injury from excessive cementing that fall outside of the curettage defect but function usually returns over time.

### Combination PMMA and allograft/bone graft substitute

Combination PMMA and allograft has not been well studied over the years. A single study met our criteria; Benevenia et al. describe the use of PMMA and allograft for curettage lesions in a small case series with 17 patients with fair results [9]. Complication included postoperative fracture and one case of progression of severe osteoarthritis. Out of our five cases, we found had no complications. Nevertheless, combination of bone filler is an area that needs to be studied more.

The use of combination PMMA and bone graft substitute has not been well described in the literature. We present three cases of using this modality without significant postoperative complications. A longer period of study is needed to understand the risks and benefits of combination methods as more long-term reports using this technique emerge.

From our results, we concluded that there was no significant difference (*P* = 0.411) in postoperative complication rates between allograft, bone graft substitute, and PMMA. Pulmonary embolus and the exceptional incidence of postoperative fracture were included during statistical analysis in our bone substitute group. We see bone graft substitute as a suitable alternative to other bone filling modalities that has generally low rates of fracture, infection, and paresthesia when compared against the literature values. When looked at individually with the Wilcoxon signed-rank test, there was no preferential type of bone filler for curettage lesions based on our allograft, bone substitute, and PMMA. The historical transition from autograft to allograft to bone graft substitute has not impacted on postoperative outcome.

Our study was limited by the retrospective nature of the study done at a single institution by one operating surgeon. Furthermore, location of curettage and type of bone filler were varied in regard to specific locations or type of lesion which can predispose to certain complications. We suggest a larger case series with comparison arms that differentiates between benign bone tumors, aggressive lesions, and metastasis, all of which existed in our case series. It is important to note that recurrence, metastatic rate, and mortality were important parameters that were outside the scope of our study. Furthermore, the degree of functional satisfaction in our study was not followed. This paper was primarily designed to focus on the complication rates involving fracture, infection, cellulitis, pulmonary embolism, and paresthesia.

The majority of skeletally immature patients received allograft or bone graft substitute. All of these patients had curettage defects that either were of significant size that led to a pathologic fracture or were considered to be an impending fracture. One patient with a large nonossifying fibroma of the proximal tibia (> 7 cm) had the defect filled with cement and years later developed a non-pathologic fracture from an 8- to 10-ft fall. The cement was subsequently removed, filled with bone graft substitute, and stabilized with a plate. The author currently uses bone cement only in subchondral defects in adults (primarily giant cell tumor) and skeletally immature children where the tumor crosses the growth plate primarily seen in chondroblastoma. Gender and size of the defect did not influence the treatment or outcome in these patients. However, patients with metastatic disease had their defects filled with bone cement because many of these patients required postoperative radiation therapy.

Some has even argued that bone defects following curettage do not require bone filling for good outcome [13, 74]. However, many structural or biologic benefits that aid in earlier return to functionality can be conferred to filling large bone defects. Although complication rates between our major bone filling modalities were comparable to each other and with the literature values, we reported late complications of large defects filled with allograft requiring reconstruction using vascularized fibular graft.

## Conclusion

In this study, we examined the outcomes after bone grafting of curettage defects. After examining 267 cases and 49 case series or cohort studies that met criteria, no statistical difference was found in complication rate between allograft, bone graft substitute, and PMMA. In the past, there has been debate over the use of bone graft substitute as a suitable bone filler for curettage defects; our studies support that it can lead to good postoperative outcome as measured by no difference in complication rate. It is possible that results may be obfuscated by a variety of bone lesion types including SBC, NOF, FD, and GCT. We see bone graft substitute as a suitable alternative to other bone filling modalities that have generally low rates of fracture, infection, and paresthesia.

## Data Availability

The datasets during and/or analyzed during the current study available from the corresponding author on reasonable request. Our patient data is deidentified, and matching identification key is no longer available. All data resources are held under lock and key at Cedars Sinai Medical Center, Los Angeles, CA, 90048, USA.

## Abbreviations

FD: Fibrous dysplasia
GCT: Giant cell tumor
NOF: Nonossifying fibroma
PMMA: Polymethyl methacrylate
SBC: Solitary bone cysts
β-TCP: β-Tricalcium phosphate
