# Correction to: Early postoperative compilations of bone filling in curettage defects

**DOI:** 10.1186/s13018-019-1425-1

**Published:** 2019-10-28

**Authors:** Clark J. Chen, Earl W. Brien

**Affiliations:** 1 Miller School of Medicine, University of Miami, Miami, FL 33136 USA; 2 Department of Orthopaedic Surgery, Cedars Sinai Medical Center, Los Angeles, CA 90048 USA

**Correction to: J Orthop Surg Res (2019) 14: 261**

**https://doi.org/10.1186/s13018-019-1297-4**

In the original publication of this article [1], there was a mistake in Fig. 2. Figure 2a and c should be swapped. The revised Fig. 2 is shown below.

**Fig. 2:**
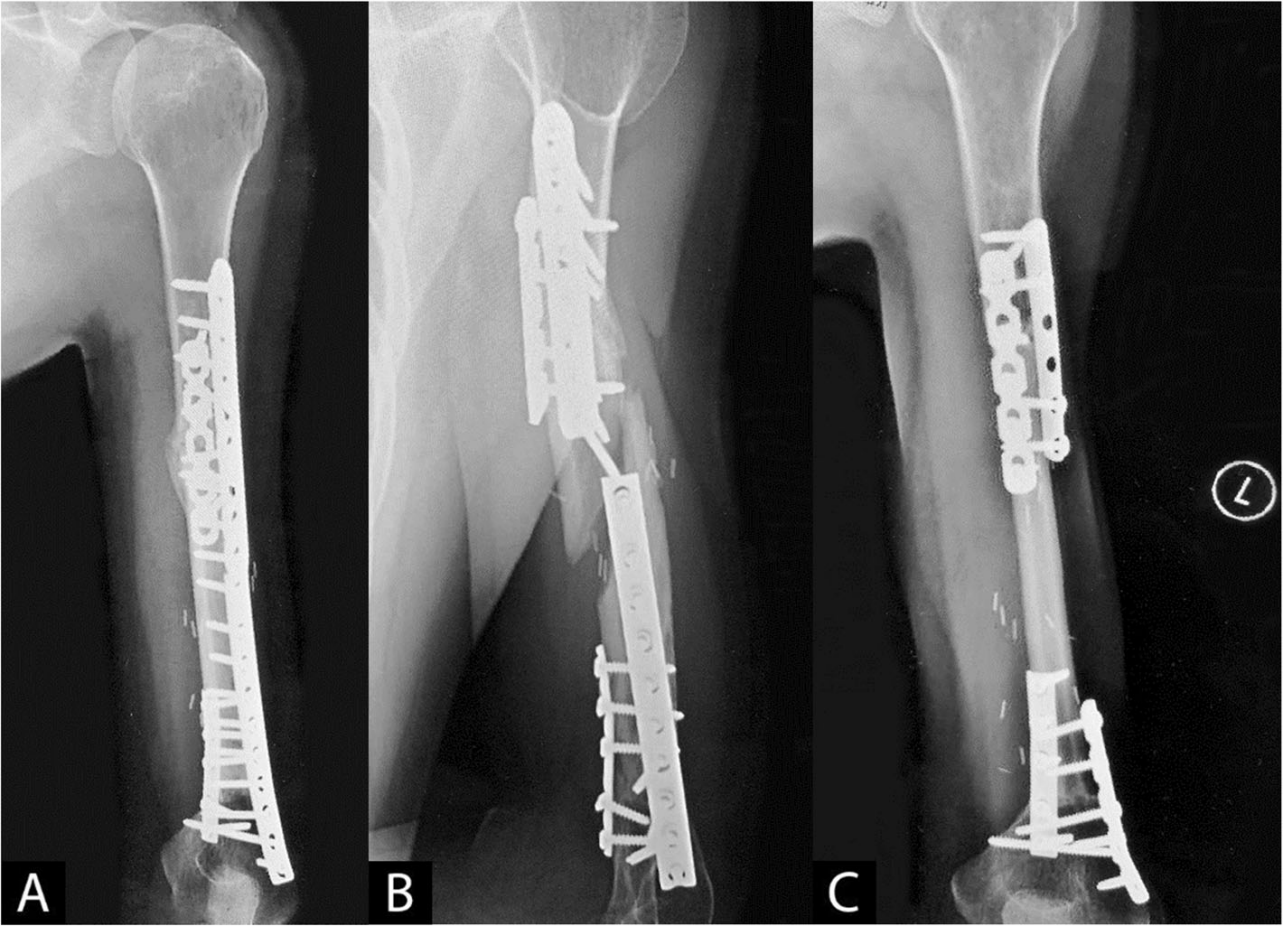
**a**–**c** Ewing’s sarcoma of the left humerus treated with chemotherapy, radiation, and resection. **a** Bulk allograft reconstruction showing ventral healing. **b** Fifteen years later, the patient suffered a fracture in his allograft from a twisting maneuver. **c** Because of the avascular central portion of the allograft, vascularized fibular autograft reconstruction was chosen, which healed well

The authors sincerely apologize for the inconvenience caused to the readers.

## References

[1] Chen CJ, Brien EW (2019). Early postoperative compilations of bone filling in curettage defects. J Orthop Surg Res.

